# Technical modifications for cost optimization in robot-assisted ventral mesh rectopexy: an initial experience

**DOI:** 10.1007/s10151-023-02756-8

**Published:** 2023-02-18

**Authors:** A. A. Marra, P. Campennì, V. De Simone, A. Parello, F. Litta, C. Ratto

**Affiliations:** 1 Proctology Unit, Fondazione Policlinico Universitario Agostino Gemelli IRCCS, Largo A. Gemelli, 8, 00168 Rome, Italy; 2 Università Cattolica del Sacro Cuore, Rome, Italy

**Keywords:** Rectal prolapse, Ventral mesh rectopexy, Robotic surgery, Cost analysis

## Abstract

**Background:**

Robot-assisted ventral mesh rectopexy is considered a valid option in the treatment of rectal prolapse. However, it involves higher costs than the laparoscopic approach. The aim of this study is to determine if less expensive robotic surgery for rectal prolapse can be safely performed.

**Methods:**

This study was conducted on consecutive patients who underwent robot-assisted ventral mesh rectopexy at Fondazione Policlinico Universitario “A. Gemelli” IRCCS, Rome, from 7 November 2020 to 22 November 2021. The cost of hospitalization, surgical procedure, robotic materials, and operating room resources in patients undergoing robot-assisted ventral mesh rectopexy with the da Vinci Xi Surgical Systems was analyzed before and after technical modifications, including the reduction of robotic arms and instruments, and the execution of a double minimal peritoneal incision at the pouch of Douglas and sacral promontory (instead of the traditional inverted J incision).

**Results:**

Twenty-two robot-assisted ventral mesh rectopexies were performed [21 females, 95.5%, median age 62.0 (54.8–70.0) years]. After an initial experience performing traditional robot-assisted ventral mesh rectopexy in four patients, we adopted technical modifications in other cases. No major complication or conversion to open surgery occurred. In total, mean cost of hospitalization, surgical procedure, robotic materials, and operating room resources was €6995.5 ± 1058.0, €5912.7 ± 877.0, €2797.6 ± 545.6, and €2608.3 ± 351.5, respectively. Technical modifications allowed a significant reduction in the overall cost of hospitalization (€6604.5 ± 589.5 versus €8755.0 ± 906.4, *p* = 0.001), number of robotic instruments (3.1 ± 0.2 versus 4.0 ± 0.8 units, *p* = 0.026), and operating room time (201 ± 26 versus 253 ± 16 min, *p* = 0.003).

**Conclusions:**

Considering our preliminary results, robot-assisted ventral mesh rectopexy with appropriate technical modifications can be cost-effective and safe.

**Supplementary Information:**

The online version contains supplementary material available at 10.1007/s10151-023-02756-8.

## Introduction

Laparoscopic ventral mesh rectopexy (LVMR), first described by D’Hoore and Penninckx [1], has been widely adopted as the treatment of choice for external or internal rectal prolapse, rectocele, and enterocele [2–5]. Use of robotic technology in performing ventral rectopexy increased to 27% in the USA from 2012 to 2015 [6]. Since its introduction [7], robot-assisted ventral mesh rectopexy (RVMR) has been a safe and effective alternative to the traditional laparoscopic technique, showing similar anatomical and functional results also in long-term follow-up [8–10]. Although RVMR did not have a clear superiority over LVMR [11], several articles reported better clinical outcomes in terms of obstructed defecation, fecal incontinence, and sexual function after robotic surgery [12–14].

Robotic technology introduced several advantages in rectal prolapse surgery, including the magnification of the three-dimensional imaging, a higher precision in the movements due to instruments with seven degrees of freedom and 90° of articulation, reduction of hand tremor, improved ergonomics for the surgeon, and a faster learning curve when compared with LVMR [10, 15–17]. Although LVMR can be exactly reproduced with the use of a robot, RVMR showed improvements in the dissection of the rectovaginal space up to the pelvic floor, the preservation of vascular and nervous pelvic structures, and placing the suture of the mesh on the ventral rectum as distally as possible in the narrow and deep space pelvis [9, 17, 18]. A trend towards a reduction in blood loss, complication rate, conversion to open surgery, and length of hospitalization has been observed in robotic surgery compared with LVMR [8, 19–21]. However, higher cost and longer operative time compared with the laparoscopic approach have reduced the initial enthusiasm, and slowed the worldwide spread of RVMR [8, 21–24].

Our hypothesis is that, when adopting the appropriate technical modifications, a less expensive RVMR can be safely performed to further enhance its treatment of rectal prolapse.

## Materials and methods

Since November 2020, the da Vinci Xi Surgical System (Intuitive Surgical, Inc., Sunnyvale, CA, USA) was adopted in rectal prolapse surgery at Fondazione Policlinico Universitario “A. Gemelli” IRCCS, an academic tertiary referral center for colorectal surgery in Rome, Italy. A prospective single-center observational study on RVMR in the surgical treatment of rectal prolapse was conducted according to the Strengthening the Reporting of Observational Studies in Epidemiology (STROBE) statement for cohort studies [25]. The study protocol was approved by our local ethics committee, and informed written consent was obtained from the patients. Consecutive patients undergoing RVMR for external or internal rectal prolapse, rectocele, and enterocele, with at least 30 days of follow-up, were considered for this study. All surgical procedures were carried out by a single surgeon (C.R.), who has previously performed about 300 open ventral mesh rectopexies.

### Data collection

During the study period we prospectively collected the following data of patients undergoing RVMR:

- *Baseline characteristics*: age, sex, body mass index (BMI), American Society of Anesthesiologists (ASA) class, previous abdominal or perineal surgery history (including hysterectomy or rectal prolapse surgery), and type of rectal prolapse before RVMR (external or internal rectal prolapse, rectocele, entero/sigmoidocele, or any association).
- *Intraoperative details*: number of robotic arms and type of instruments adopted, total operative time (defined as surgical time from skin incision to wound closure), initial laparoscopic phase, robotic docking and surgeon robot console time, conversion to open surgery, blood loss (if greater than 20 ml), and intraoperative complications.
- *Perioperative data*: length of hospital stay, postoperative complications (classified according to the Clavien–Dindo classification), and early recurrences of rectal prolapse at follow-up visit and clinical examination routinely performed at 30 days from surgery.

Cost analysis was recovered by the health management of our institution, investigating cost of hospitalization, surgical procedure, robotic materials, and operating room resources for each patient undergoing RVMR, before and after adopting technical modifications, as detailed below. Cost of hospitalization included the total expenses incurred for each single hospitalization. All robotic and surgical procedure costs (e.g., mesh, trocars, and other single-use materials) were defined as surgical procedure costs. However, the impact of robot-related expenses and materials on total costs of hospitalization were separately evaluated. Operating room resources were defined as costs related to the use of the operating theater, including healthcare personnel and equipment. Costs regarding hospital stay (including pre- and postoperative therapies) and laboratory examinations were considered separately.

### Modifications of the surgical techniques

**Fig. 1:**
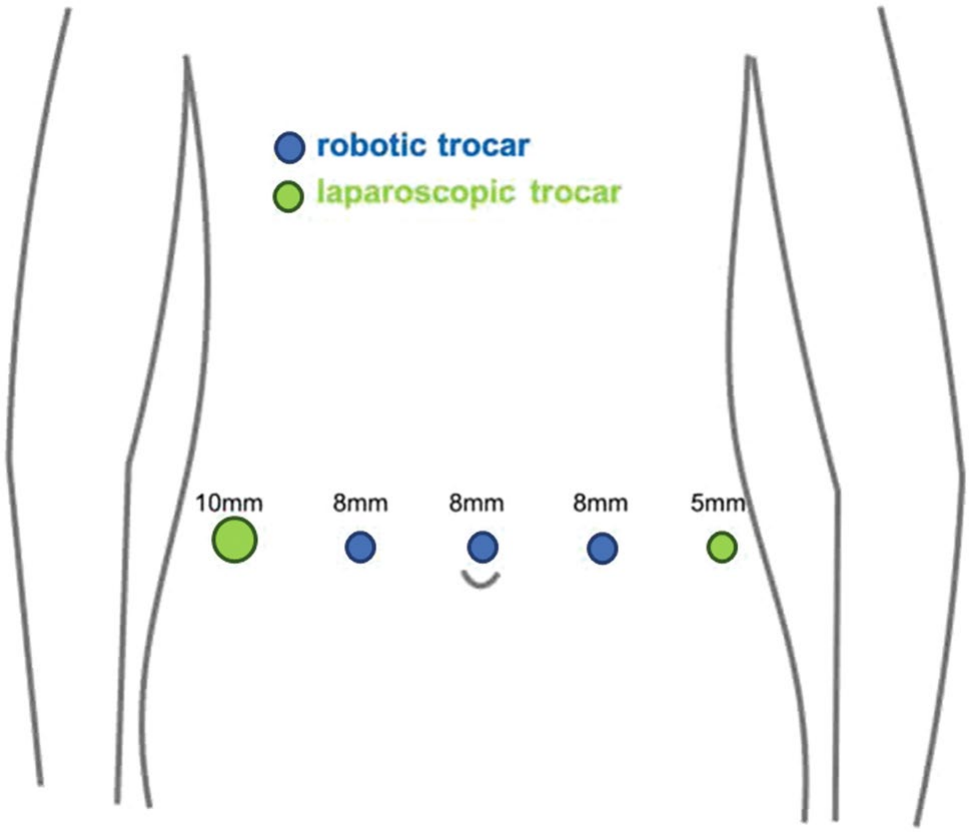
Trocar positions after technical modifications

**Fig. 2:**
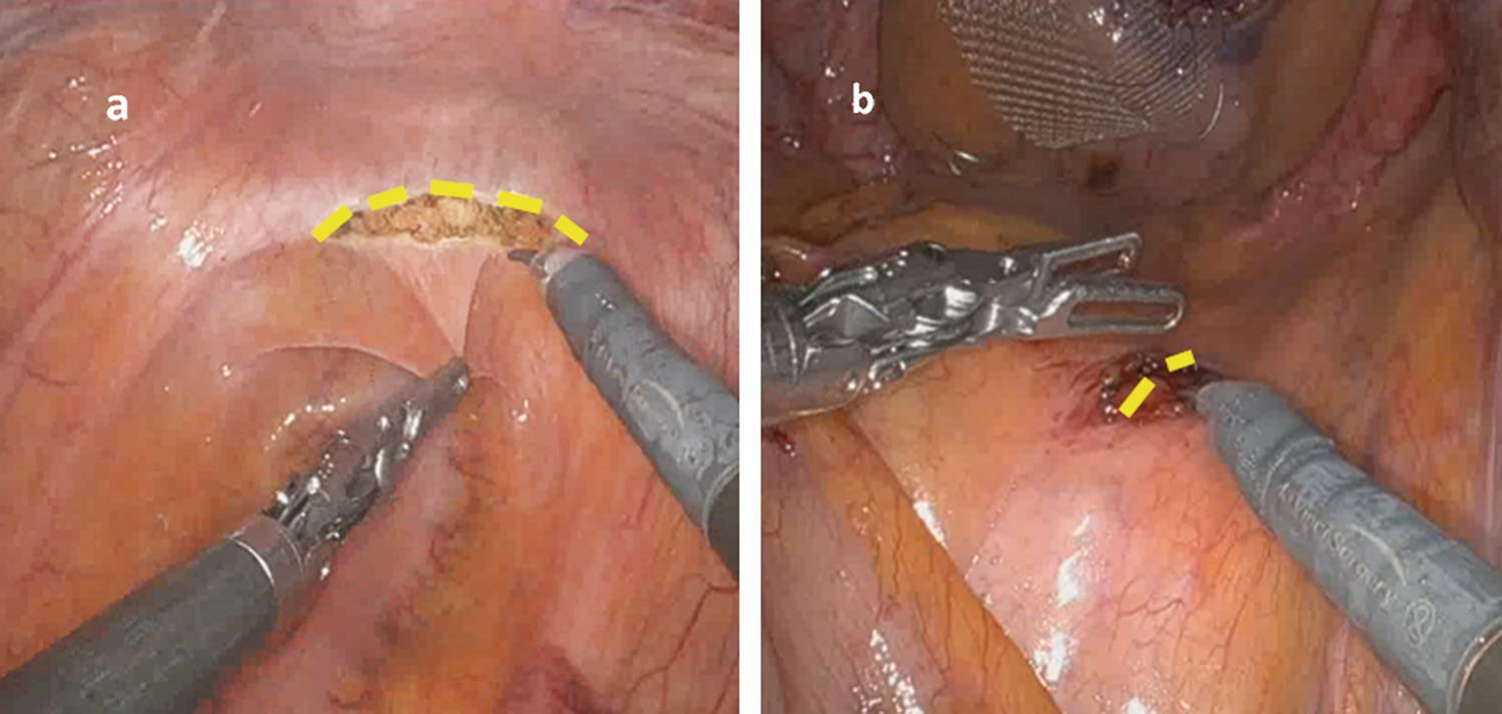
Two peritoneal incisions were performed at the pouch of Douglas (**a**) and sacral promontory (**b**)

**Fig. 3:**
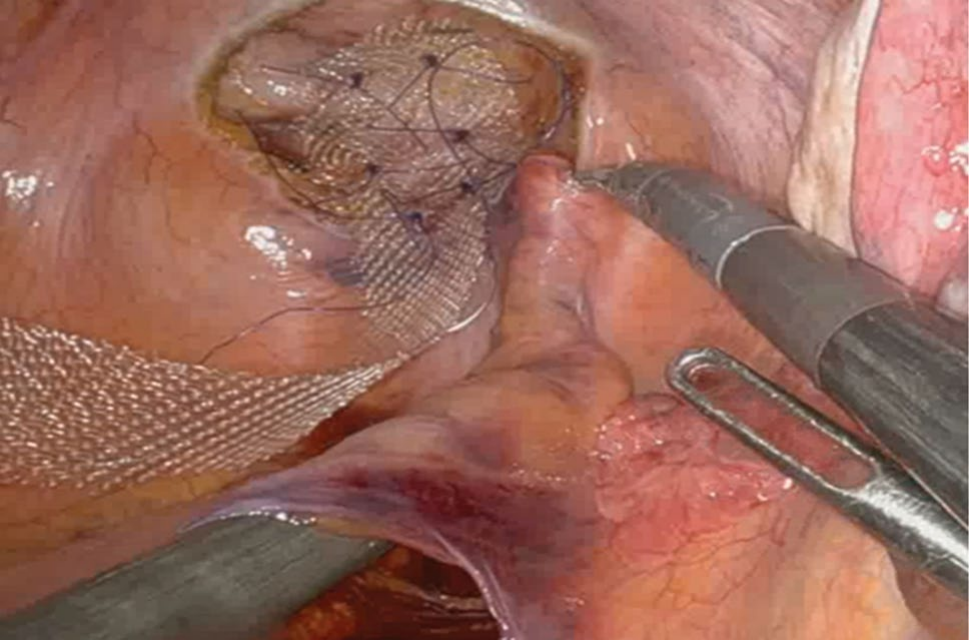
A retroperitoneal tunnel was gently created along the right side of the rectum, paying attention to the pelvic structures

**Fig. 4:**
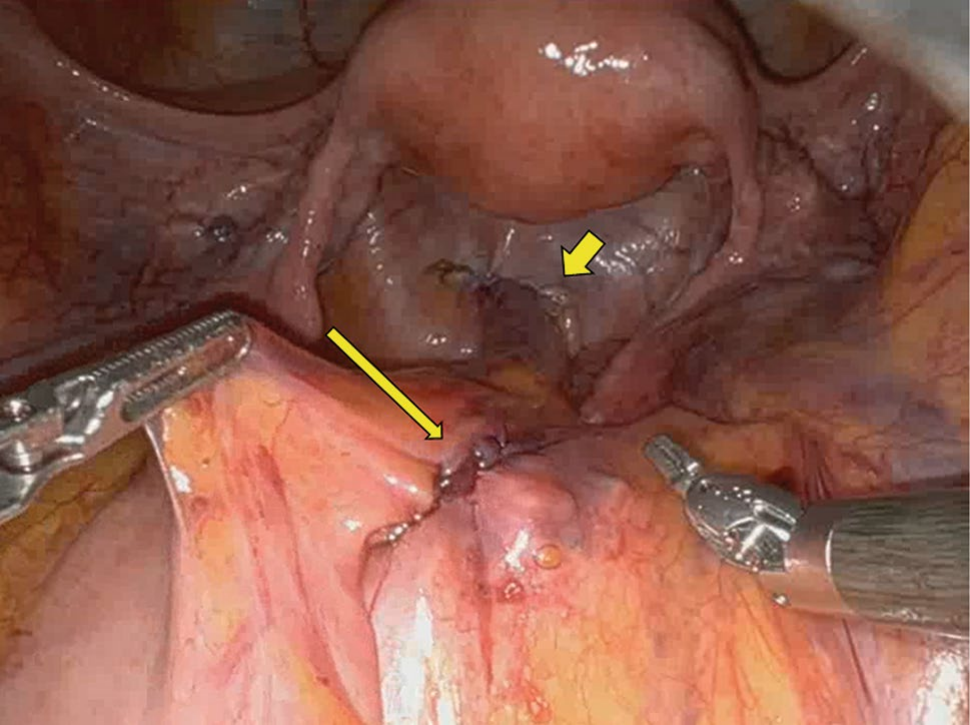
Peritoneal incisions at the pouch of Douglas (short arrow) and sacral promontory (long arrow) were sutured with two continued V-Loc sutures

After an initial experience performing the traditional procedure of RVMR using four robotic arms, without any limitation of the available robotic instruments, we introduced several modifications to the technique, as shown in our previous video vignette [26] to optimize robot-related costs. Briefly, modified RVMR minimized the number of robotic arms and instruments without any substantial changes in the execution of the surgical procedure. Robotic arms and ports were reduced from four (as traditionally used) to three, and laparoscopic assistance was intensified (two ports), allowing for the “controlled” traction of sigmoid colon to the left side of the abdomen (by the same assistant at the operating table) during the rectovaginal dissection, and fixation of the mesh at the sacral promontory (Fig. 1). Robotic instruments were also revised: we used only robotic Cadiere forceps, monopolar curved scissors, and a large needle driver. Other instruments (e.g., a robotic fenestrated bipolar forceps) were not routinely utilized. In female patients, an intrauterine manipulator was preferred to hitching the uterus to the abdominal wall with a suture to manipulate the uterovaginal structures more effectively during the rectovaginal space dissection, as well as reducing the risk of uterine bleeding. Any intraperitoneal adhesiolysis was carried out either laparoscopically, or robotically. After docking of robotic instrument to the ports with patient in the Trendelenburg position, the robotic procedure started with small peritoneal incisions at the apex of the pouch of Douglas and the sacral promontory (instead of the traditional inverted J incision along the right side of the rectal wall Fig. 2). Through the peritoneal incision at the pouch of Douglas, the rectovaginal space was dissected up to the perineal body. A polypropylene mesh was fixed to the most distal ventral aspect of the rectum, with three 3-0 PDS sutures. The second small incision at the level of the sacral promontory exposed the sacral periosteum. Thereafter, if it was technically feasible and safe, a retroperitoneal tunnel was created from the sacral promontory to the Douglas incision, along the right side of the rectum, as showed in Fig. 3. This step was adopted to avoid injury to the support structures of the rectum (i.e., the right uterosacral ligament and the right lateral ligament of the rectum). The proximal edge of the mesh was then pulled up to the sacral promontory and, under a gentle tension, fixed with two 2-0 PDS sutures. The posterior vagina, at the level of posterior fornix, was approximated to the mesh and the ventral rectum to prevent a residual enterocele. Finally, the peritoneal incisions were closed with two continuous V-Loc sutures (Fig. 4).

### Statistical analysis

Data are presented as mean and standard deviation, or frequency and percentages for continuous and categorical variables, respectively. Pearson’s chi-squared test and Mann–Whitney *U* nonparametric test were used due to skewed distribution to assess categorical and continuous data in patients who underwent traditional versus modified RVMR. Missing data were excluded from analysis. A *p* value < 0.05 was considered statistically significant. Statistical analysis was performed with IBM SPSS Statistics for Windows, Version 25.0 (IBM Corp, Armonk, NY, USA).

## Results

Twenty-two patients [21 females, 95.5%, median age 62.0 (54.8–70.0) years] had RVMR, completed the 30-day follow-up period, and were included in the study. After the initial experience performing traditional RVMR (including the inverted-J peritoneal incision) in the first four female patients, we adopted technical modifications in the last 18 cases (17 females, 94.4%). No missing data were observed.

Preoperative patient characteristics are provided in Table 1. Mean total operative time was 211 ± 32 min, with an initial laparoscopic phase, a robotic docking, and a surgeon robot console time of 30 ± 7, 15 ± 5, and 150 ± 35 min, respectively. No conversion to open surgery occurred. Mean blood loss was not assessable because it was generally minimal. One female case in the modified robotic cohort, where bleeding from a giant pelvic varicocele occurred, was managed successfully using a robotic fenestrated bipolar forceps, with a total blood loss of about 100 ml. No other treatment was required, and the patient was discharged on postoperative day 2 with stable hemoglobin levels. Postoperatively, one catheter-related urinary infection requiring antibiotic therapy (classified as a grade II of the Clavien–Dindo classification) occurred in the group of patients that underwent RVMR before technical modifications. Mean length of hospital stay was 2.2 ± 0.4 days. No major complications, early recurrences of rectal prolapse, or reoperations occurred within 30 days after surgery.

**Table 1:** Baseline patients’ characteristics

	No. (%)
Patients	22
Ratio F:M	21:1
Age (years)*	60.3 ± 14.0
BMI (kg/m^2^)*	23.6 ± 4.4
ASA class	
I	1 (4.5)
II	19 (86.4)
III	2 (9.1)
Type of rectal prolapse	
External rectal prolapse	4 (18.2)
Internal rectal prolapse	18 (81.8)
Rectocele (mm)*	49.1 ± 13.1
Entero/sigmoidocele	10 (45.5)
Previous abdominal surgery	13 (59.1)
Previous hysterectomy	3 (13.6)
Previous perineal surgery	7 (31.8)
Previous rectal prolapse surgery	3 (13.6)

*No.* number, *F *female, *M* male, *BMI* body mass index, *ASA* American Society of Anesthesiologists

*Data are shown as mean ± standard deviation

The mean costs of hospitalization, surgical procedure, robotic materials, and operating room resources were €6995.5 ± 1058.0, €5912.7 ± 877.0, €2797.6 ± 545.6, and €2608.3 ± 351.5, respectively. In particular, robot-related costs accounted for 40.0% of the total hospitalization costs. Mean hospital stay (including pre- and postoperative therapies) and laboratory examination costs were € 901.8 ± 234.5 and €181.0 ± 62.0, respectively.

### Comparison of RVMR before and after technical modifications

No differences in baseline characteristics were noted in the patients undergoing traditional procedure versus modified RVMR. As presented in Table 2, technical changes in RVMR allowed a significant reduction in overall hospitalization, surgical procedure, robotic materials, and operating room resources costs. Other costs regarding hospital stay and laboratory exams were no different between the two groups. Furthermore, after adopting technical modifications in RVMR, we observed a reduction in the number of robotic instruments, surgeon robot console time, and total operative time. Length of hospital stay was slightly reduced in patients who underwent modified RVMR (although the differences were not statistically significant). Conversely, no differences were observed in intra- and perioperative complications, conversion rate, initial laparoscopic phase, and robotic docking time.

**Table 2:** Comparison between data collected in patients who underwent robot-assisted ventral mesh rectopexy before and after technical modifications (Mann–Whitney *U* and chi-squared tests)

	Before no. (%)	After no. (%)	*p* value
RVMR performed	4	18	
Robotic arms (units)	4	3	
Robotic instruments (units)*	4.0 ± 0.8	3.1 ± 0.2	**0.026**
Operating room times (min)*			
Initial laparoscopic phase	30 ± 7	30 ± 7	0.967
Robotic docking	15 ± 4	15 ± 5	0.837
Surgeon robot console	208 ± 16	138 ± 23	**0.001**
Total operative time	253 ± 16	201 ± 26	**0.003**
Conversion to open surgery	0 (0)	0 (0)	–
Intraoperative complications	0 (0)	1 (5.6)	0.818
Postoperative complications	1 (25)	0 (0)	0.182
Length of hospital stay (days)*	2.8 ± 0.5	2.1 ± 0.3	0.053
Cost analysis (euros)*			
Overall hospitalization	8755.0 ± 906.4	6604.5 ± 589.5	**0.001**
Surgical procedure	7426.6 ± 831.3	5576.3 ± 411.2	**0.001**
Robotic materials	3728.8 ± 660.2	2590.7 ± 203.5	**0.001**
Operating room resources	3065.4 ± 269.7	2506.8 ± 282.3	**0.003**
Hospital stay (including therapies)	1120.0 ± 184.8	853.3 ± 219.5	0.066
Laboratory examinations	208.4 ± 40.8	174.9 ± 65.1	0.538

*p* values < 0.05 is highlighted in bold

*No.* number, *RVMR* robot-assisted ventral mesh rectopexy

*Data are shown as mean ± standard deviation

## Discussion

Several technical variations to the standard LVMR have been previously described in the literature [20], the main one being the robotic approach, which provided precision, easy execution, and freedom of movement typical of robotic instruments [27, 28]. In this study utilizing RVMR, we assessed the feasibility of further technical modifications of the traditional procedure, and the impact on the costs of robotic surgery. Although there was one case of bleeding, which was successfully managed intraoperatively, our technical changes did not significantly increase the intra- and postoperative complications. Moreover, no conversions to open surgery, reoperations, or early recurrences were observed with the modified technique.

Although some suggest RVMR is associated with improved long-term quality of life compared with LVMR, thus justifying the overall cost [29], costs related to robotic surgery are still substantial [8, 21–24]. However, assessment of the costs reported in the literature, and in our study, are limited by heterogeneity in the reimbursement policy adopted by any national health system, or agreements between the hospital administration, and any company producing robotic technologies. Nevertheless, we showed that adequate modifications in the surgical technique allow a significant reduction in overall cost of hospitalization. Accordingly, the uptake of RVMR may be enhanced, mainly in referral centers where the robot is routinely used in colorectal surgery and the costs of purchase and maintenance are already covered. Moreover, modified RVMR could become increasingly accessible and cost-effective in the future, especially considering the rapid evolution of robotic surgery and technologies (e.g., the increase of robotic instrument life, or the introduction of a portable and cheaper robotic system competitor) [12, 18, 30].

In our study, the number of robotic arms and instruments was simply optimized to reduce any unnecessary cost. As already observed in the literature [11], we soon noticed that the fourth arm had no real surgical benefit. Therefore, even if additional instruments were available in the operating room, only three robotic arms and instruments were routinely provided and used for each operation.

A reduction in mean operative time was observed with our technical changes (i.e., the reduction of robotic arms and instruments, the minimal peritoneal incisions, and the retroperitoneal tunnel), despite including all RVMR patients, even those early in our learning curve [17, 18]. This inclusion of all patients perhaps explains why the total operative time was longer than reported in other studies [8]. Nevertheless, we believe that the time spent creating a retroperitoneal tunnel, and the suturing the two small peritoneal incisions, is less than the time spent incising and suturing the longer peritoneal J-inverted incision as described in the traditional LVMR. We expect our total operating time will decrease further with increased experience [23, 31].

Although our data did not show a statistically significant difference in length of hospital stay with a robotic approach, the trend toward a shorter stay is consistent with data of robotic surgery in general [8, 21]. We are confident that modified RVMR could also be adapted to a day case surgery setting [32].

Limitations of this preliminary study should be addressed. Although we demonstrated a significant reduction of costs associated with RVMR, the sample size was small. Unfortunately, in the past few years (mainly due to COVID-19 pandemic), the number of robotic sessions has been significantly reduced. We are hopeful this will be reversed in the future. The introduction of new robotic platforms may have a substantial influence on costs, but we did not account for this in this study. We also did not account for other cost-saving technical aspects, such as hitching the uterus and the sigmoid colon with a simple suture or tack. While this has been described, we prefer a intrauterine manipulator for its versatility, as detailed above. Out paper did not report functional and quality of life outcomes. We plan to report on these variables after a longer follow-up period. Finally, the costs related to preoperative assessment and follow-up visits were not included. We would expect these to be consistent, regardless of intervention technique.

## Conclusions

These preliminary results showed that technical modifications can reduce costs in the robotic treatment of rectal prolapse. This is particularly likely in centers where robotic surgery is routinely performed. Further comparative and multicenter studies evaluating long-term outcomes related to cost-analysis are needed to confirm our preliminary results. A reduction of robot-related purchase and maintenance costs, an improvement of the dedicated robotic team experience, and the evaluation of further long-term results in RVMR are our future goals.

## Supplementary Information

Below is the link to the electronic supplementary material.

Supplementary file1 (DOCX 34 KB)

## Data Availability

All data, analytic methods, and study materials used to conduct the research will be made available to any researcher from the corresponding author (CR), upon reasonable request.

## References

[1] D'Hoore A, Cadoni R, Penninckx F (2004). Long-term outcome of laparoscopic ventral rectopexy for total rectal prolapse. Br J Surg.

[2] Faucheron JL, Trilling B, Girard E, Sage PY, Barbois S, Reche F (2015). Anterior rectopexy for full-thickness rectal prolapse: technical and functional results. World J Gastroenterol.

[3] van Iersel JJ, Paulides TJ, Verheijen PM, Lumley JW, Broeders IA, Consten EC (2016). Current status of laparoscopic and robotic ventral mesh rectopexy for external and internal rectal prolapse. World J Gastroenterol.

[4] Formijne Jonkers HA, Poierrié N, Draaisma WA, Broeders IA, Consten EC (2013). Laparoscopic ventral rectopexy for rectal prolapse and symptomatic rectocele: an analysis of 245 consecutive patients. Colorectal Dis.

[5] Consten EC, van Iersel JJ, Verheijen PM, Broeders IA, Wolthuis AM, D'Hoore A (2015). Long-term outcome after laparoscopic ventral mesh rectopexy: an observational study of 919 consecutive patients. Ann Surg.

[6] Damle A, Damle RN, Flahive JM (2017). Diffusion of technology: trends in robotic-assisted colorectal surgery. Am J Surg.

[7] Munz Y, Moorthy K, Kudchadkar R (2004). Robotic assisted rectopexy. Am J Surg.

[8] Bao X, Wang H, Song W, Chen Y, Luo Y (2021). Meta-analysis on current status, efficacy, and safety of laparoscopic and robotic ventral mesh rectopexy for rectal prolapse treatment: can robotic surgery become the gold standard?. Int J Colorectal Dis.

[9] Laitakari KE, Mäkelä-Kaikkonen JK, Pääkkö E (2020). Restored pelvic anatomy is preserved after laparoscopic and robot-assisted ventral rectopexy: MRI-based 5-year follow-up of a randomized controlled trial. Colorectal Dis.

[10] Mäkelä-Kaikkonen J, Rautio T, Pääkkö E, Biancari F, Ohtonen P, Mäkelä J (2016). Robot-assisted vs laparoscopic ventral rectopexy for external or internal rectal prolapse and enterocele: a randomized controlled trial. Colorectal Dis.

[11] Faucheron JL, Trilling B, Girard E (2019). Robotic ventral mesh rectopexy for rectal prolapse: a few years until this becomes the gold standard. Tech Coloproctol.

[12] Mantoo S, Podevin J, Regenet N, Rigaud J, Lehur PA, Meurette G (2013). Is robotic-assisted ventral mesh rectopexy superior to laparoscopic ventral mesh rectopexy in the management of obstructed defaecation?. Colorectal Dis.

[13] Mäkelä-Kaikkonen J, Rautio T, Kairaluoma M (2018). Does ventral rectopexy improve pelvic floor function in the long term?. Dis Colon Rectum.

[14] Laitakari KE, Mäkelä-Kaikkonen JK, Kössi J (2022). Mid-term functional and quality of life outcomes of robotic and laparoscopic ventral mesh rectopexy: multicenter comparative matched-pair analyses. Tech Coloproctol.

[15] Ayav A, Bresler L, Hubert J, Brunaud L, Boissel P (2005). Robotic-assisted pelvic organ prolapse surgery. Surg Endosc.

[16] Corcione F, Esposito C, Cuccurullo D (2005). Advantages and limits of robot-assisted laparoscopic surgery: preliminary experience. Surg Endosc.

[17] van der Schans EM, Verheijen PM, Moumni ME, Broeders IAMJ, Consten ECJ (2022). Evaluation of the learning curve of robot-assisted laparoscopic ventral mesh rectopexy. Surg Endosc.

[18] Perrenot C, Germain A, Scherrer ML, Ayav A, Brunaud L, Bresler L (2013). Long-term outcomes of robot-assisted laparoscopic rectopexy for rectal prolapse. Dis Colon Rectum.

[19] Mehmood RK, Parker J, Bhuvimanian L (2014). Short-term outcome of laparoscopic versus robotic ventral mesh rectopexy for full-thickness rectal prolapse. Is robotic superior?. Int J Colorectal Dis.

[20] Albayati S, Chen P, Morgan MJ, Toh JWT (2019). Robotic vs. laparoscopic ventral mesh rectopexy for external rectal prolapse and rectal intussusception: a systematic review. Tech Coloproctol.

[21] Ramage L, Georgiou P, Tekkis P, Tan E (2015). Is robotic ventral mesh rectopexy better than laparoscopy in the treatment of rectal prolapse and obstructed defecation? A meta-analysis. Tech Coloproctol.

[22] Heemskerk J, de Hoog DE, van Gemert WG, Baeten CG, Greve JW, Bouvy ND (2007). Robot-assisted vs. conventional laparoscopic rectopexy for rectal prolapse: a comparative study on costs and time. Dis Colon Rectum.

[23] Mäkelä-Kaikkonen J, Rautio T, Klintrup K (2014). Robotic-assisted and laparoscopic ventral rectopexy in the treatment of rectal prolapse: a matched-pairs study of operative details and complications. Tech Coloproctol.

[24] Rondelli F, Bugiantella W, Villa F (2014). Robot-assisted or conventional laparoscoic rectopexy for rectal prolapse? Systematic review and meta-analysis. Int J Surg.

[25] von Elm E, Altman DG, Egger M (2008). The Strengthening the Reporting of Observational Studies in Epidemiology (STROBE) statement: guidelines for reporting observational studies. J Clin Epidemiol.

[26] Ratto C, Marra AA, Campennì P, De Simone V, Litta F, Parello A (2022). Modified robotic ventral mesh rectopexy—a video vignette. Colorectal Dis.

[27] Naldini G, Fabiani B, Sturiale A, Russo E, Simoncini T (2021). Advantages of robotic surgery in the treatment of complex pelvic organs prolapse. Updates Surg.

[28] Postillon A, Perrenot C, Germain A (2020). Long-term outcomes of robotic ventral mesh rectopexy for external rectal prolapse. Surg Endosc.

[29] Mäkelä-Kaikkonen J, Rautio T, Ohinmaa A (2019). Cost-analysis and quality of life after laparoscopic and robotic ventral mesh rectopexy for posterior compartment prolapse: a randomized trial. Tech Coloproctol.

[30] Jensen CC, Madoff RD (2016). Value of robotic colorectal surgery. Br J Surg.

[31] Mantoo S, Rigaud J, Naulet S, Lehur PA, Meurette G (2014). Standardized surgical technique and dedicated operating room environment can reduce the operative time during robotic-assisted surgery for pelvic floor disorders. J Robot Surg.

[32] Faucheron JL, Trilling B, Barbois S, Sage PY, Waroquet PA, Reche F (2016). Day case robotic ventral rectopexy compared with day case laparoscopic ventral rectopexy: a prospective study. Tech Coloproctol.

